# Assessing the Distribution and Richness of Mammalian Species Using a Stacking Species Distribution Model in a Temperate Forest

**DOI:** 10.3390/ani14050759

**Published:** 2024-02-29

**Authors:** Ok-Sik Chung, Jong Koo Lee

**Affiliations:** 1 Space and Environment Laboratory, Chungnam Institute, 73-26 Institute Road, Gongju 32589, Republic of Korea; nansamata@hanmail.net; 2 Division of Life Sciences, College of Life Sciences and Bioengineering, Incheon National University, 119 Academy-ro, Yeonsu-gu, Incheon 22012, Republic of Korea

**Keywords:** habitat suitability, species richness mapping, macroecological models, conservation

## Abstract

**Simple Summary:**

This study examined the question of how many different mammalian species reside in the Province of Chungnam, Korea, and the influence of environmental, climatic, and human factors. A special model that combines the distribution of each species was used. Distance to forest boundary, elevation, slope, human population density, and distance to water streams were the major factors in determining overall species richness, while climate factors showed relatively less importance. Of particular interest was the fact that the map of unique species aligned with the location of many mammal species. The model would be very helpful to land managers, as well as those who are seeking a method for accommodating greater species diversity.

**Abstract:**

This study was conducted as an effort to examine the association between mammalian species richness and environmental, anthropogenic, and bioclimate factors in the Province of Chungnam, Korea, using a stacked species distribution model (SSDM) approach. An SSDM model was constructed using an extensive dataset collected from 1357 mammal sampling points and their corresponding forest, geographical, anthropogenic, and bioclimatic information. Distance to forest edge, elevation, slope, population density, and distance to water channels were identified as important variables for determining species richness, whereas the impact of bioclimate variables was less important. The endemism map showed a strong correlation with species richness, suggesting the important role of endemic species. Overestimation was observed in areas with lower species richness. However, the findings of the study still demonstrated that valuable insights can be obtained through the use of the SSDM, which may be helpful to land managers, aiding in the effective management of wildlife habitats, particularly in regions with an abundance of species richness and endemism.

## 1. Introduction

In recent decades, species distribution models (SDMs) have been utilized as essential tools for understanding the suitability of current habitats and evaluating biological responses to specific or changing environmental factors [1]. Increased numbers of large datasets with spatial information, incorporating diverse species, and advances in computing capabilities and modeling techniques have led to increased efforts toward the development of SDMs [2,3]. Thus, various SDMs using different modeling/machine learning approaches, including generalized linear models (GLMs), generalized additive models (GAMs), multivariate adaptive regression splines (MARSs), artificial neural networks (ANNs), classification and regression trees (CARTs), maximum entropy (MaxEnt), gradient boosting machine (GBM), and random forest (RF), have been suggested and applied to many regions and taxonomy [4].

However, coincident forecasts are not always obtained when using SDMS; they often yield contrasting predictions [1]. These discrepancies can result from differences in model parameterizations, model selection criteria, and data characteristics (e.g., sample size, scale, and correlations between the environmental variables [5]). However, consistent superior performances across diverse species and regions have not yet been reported for a single novel method [5,6,7].

To address this challenge, a proposed solution involving the development of a consensus by combining outputs from multiple SDMs (termed “ensembles”) has been suggested [8]. Each SDM contains both true signals representing the relationships that the model intends to capture and the false noise generated by errors and uncertainties inherent to the data and model structure [5]. It is anticipated that the process of combining will result in the enhanced segregation of true signals [8].

The aggregation and extrapolation of multiple SDMs can be regarded as a promising measure for the assessment of site- or community-level biodiversity [9]. The process of combining is often called “stacking”; thus, such models are also referred to as stacked species distribution models (SSDMs, e.g., [3,10]). Valuable insights into the spatial distribution patterns of biodiversity can be obtained through the use of SSDMs, providing scientists and decision makers with information that is more accessible and interpretable [9].

Considering the significance of species richness as a simple yet substantial metric in assessing biodiversity [11], identifying regions with high species richness (e.g., richness maps) can be critical in the effort to develop strategies that can be used for the effective management and conservation of habitats. However, capturing sufficient variation in species richness across large areas with comprehensive inventories is always challenging [12], often due to insufficient or inadequate planning of data collection efforts [13]. Particularly in Korea, the structure and organization of communities, including the mammalian community, are largely unknown and lack statistical/quantitative robustness.

Therefore, the objective of this study was to examine the associations between mammalian species richness and various environmental, anthropogenic, or bioclimatic variables using a stacked species distribution approach. An additional objective of this study was to identify specific areas where high species richness is predicted within the Province of Chungnam. If the results of the study can be extrapolated to other species (e.g., [14]), the findings of this study will provide valuable insights that may be useful to land managers, supporting their decisions toward more effective strategies that can be used for the management of wildlife habitats and land.

## 2. Materials and Methods

### 2.1. Study Area

**Figure 1:**
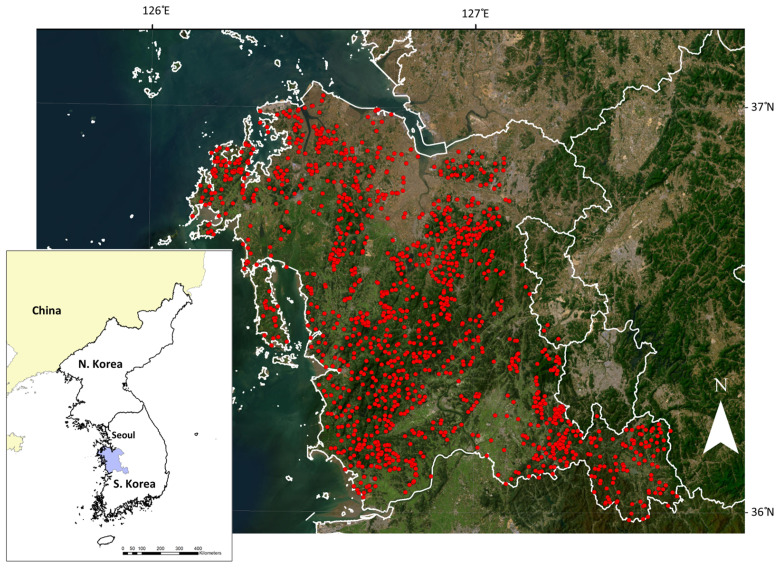
The Province of Chungnam (marked light blue in bottom left panel) and the location of the sampling points (red dots).

This study was conducted across the Province of Chungcheongnam (“Chungcheongnam-do” or “Chungnam”; hereafter referred to as Chungnam), which is located in the mid-western part of South Korea (35°00′~37°06′ N, 126°13′~127°63′ E; Figure 1). Chungnam covers an area of 8226 km^2^, consisting primarily of hilly or lowland terrain [15]. Over 70% of the land consists of forests and agricultural lands [16], and most forests have been reforested since the 1970s, featuring a mix of coniferous, deciduous, and mixed forests [17]. The average stocking volume of these forests was 139 m^3^/ha as of 2018 [18].

The humid continental climate of the study area is characterized by hot and humid summers and dry and cold winters [19]. The average annual temperature is 12.2 °C; the highest monthly temperature is 25.3 °C in August, and the lowest temperature is 1.7 °C in January. The average annual precipitation is 1310 mm, primarily concentrated in summer (July–September) [20].

### 2.2. Data and Analysis

The collection of extensive mammal survey data was performed during the Biotope Mapping Project (2008–2014; Chungnam Institute) in the Province of Chungnam. The project was conducted for the purpose of classifying and identifying biotopes across the province; 1483 sampling points were randomly assigned. The field campaign also included an extensive wildlife survey (>~3500 survey points) across the province through 2–3 phases [21]. For the mammal survey, the researchers performed a thorough examination of the trace of mammal species within a 50 m radius at each sampling point. One to three skilled surveyors systematically established transects with 5–10 m intervals across the sampling area. They traversed these transects while recording the presence of all mammalian species through direct observations or the detection of signs such as tracks, feeding signs, habitats, scat, hair/fur, and carcasses. In this study, 1357 mammal sampling points randomly located in the forests were used for analysis (Figure 1).

Information relating to the forests in the sampling points was derived from the Korea Forest Cover Type Map (Korea Forest Service, Daejeon, Republic of Korea). The extracted information on forests included forest area (F_AREA), tree diameter class (DIA_CL), and the distance to the nearest forest edge (DIST_FOR; Table 1). The tree diameter class was categorized as median diameter at breast height (DBH) within the stand: (>50% of total trees were) <6 cm as size class 1, 6–18 cm as size class 2, 18–30 cm as size class 3, and >30 cm as size class 4, respectively.

Geographical and anthropogenic variables were extracted from digital thematic maps provided by the Korea National Geographic Information Institute (Suwon, Republic of Korea). A digital elevation model, road network, water channel maps, and population data were used to obtain the distance to the water channel (DIST_WAT), elevation (ELEV), population density (POP_DEN), and road density (RD_DEN) for each wildlife sampling point (Table 1).

Bioclimatic information for each sampling point was obtained from historical climate data from the WorldClim database (WorldClim.org (accessed on 15 October 2023), ver. 2.1; [22]). Nineteen standard bioclimate variables represent the averages for 1970–2000 with 30 s resolution. Details regarding the bioclimate variables are shown in Table 1.

All procedures for the processing and mapping of spatial data were performed at a resolution of 100 m × 100 m (except bioclimate variables) using ArcGIS Desktop (ver. 10.8.1 ESRI Inc. Redlands, CA, USA).

The ensemble species distribution model (ESDM) for each species of mammal was fitted against the 27 variables (Table 1) using a generalized linear model (GLM; [23]), generalized boosted regression model (GBM [24]), random forest (RF [25]), MaxEnt (MAXENT [26]), classification tree analysis (CTA [27]), and support vector machines (SVMs [28]). Each algorithm was implemented and evaluated using a hold-out cross-validation method. Seventy percent of the data were partitioned into a training set, and the cross-validation process was iterated twice per algorithm. All of the other settings for the individual algorithms were tuned to the defaults specified by Schmitt et al. [12]. The area under the receiver operating characteristic curve, with a selection threshold set to 0.6, was used to select the algorithms for the ensemble process.

The stacked species distribution model (SSDM) was generated to evaluate species richness by aggregating continuous habitat suitability maps for the ESDM for each species [29]. The staking process employed the spatially explicit species assemblage modeling (SESAM) framework [30]. The SESAM framework refines species richness prediction through four consecutive filtering processes: (1) dispersal filtering, (2) habitat suitability filtering using SDMs, (3) incorporating macroecological constraints through macroecological models, and (4) biotic filtering through ecological assembly rules [12,30].

The importance of environmental variables was analyzed in two steps. First, each ESDM (full model) was compared with a refitted ESDM that omitted the target environmental variable (reduced model). The Pearson’s correlation coefficient (*r*) for predictions between the full model and the reduced model was computed, and the score for each environmental variable was calculated as 1-*r*. Subsequently, these scores were averaged across all ESDMs [12].

Additionally, an endemism map was constructed to explore the location where a unique species occurs within the study area. This involved the weighted endemism index by scoring endemism (i.e., counting species richness) in a cell while weighing each species by the inverse of its range [31].

The assessment of the performance of SSDM was based on (1) the species richness error, (2) assemblage prediction success, (3) Cohen’s kappa, (4) specificity, (5) sensitivity, and (6) the Jaccard index [32], as suggested by Schmitt et al. [12]. In addition, a simple macroecological model (i.e., Poisson regression fitting species richness as a dependent variable) was fitted and compared using a scatter plot for the evaluation of the overprediction issue. All model constructions (ESDMs and an SSDM) and assessments were performed using the SSDM package [12] of R statistical software [23].

## 3. Results

The field campaign identified 4414 traces from 16 mammal species found throughout the province. Korean water deer (*Hydropotes inermis*) was observed most frequently, followed by large mole (*Mogera robusta*), Eurasian red squirrel (*Sciurus vulgaris*), common raccoon dog (*Nyctereutes procyonoides*), and leopard cat (*Prionailurus bengalensis*), respectively (Table 2).

**Figure 2:**
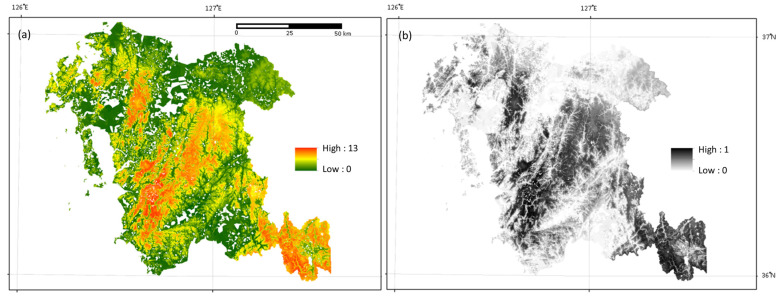
(**a**) Mammalian species richness and (**b**) endemism maps of the Province of Chungnam, predicted using a stacked species distribution model.

The local species richness of mammalian species estimated using the SSDM ranged from 0.3 to 12.6 (average: 4.4). High species richness was observed in regions dispersed in southwestern directions, along with mountain ranges such as the Charyeong and Noryeong mountain ranges (Figure 2a). The pattern shown on the endemism map was similar to that observed on the local species richness map, suggesting a trend toward greater mammalian species richness in regions with high endemism (Figure 2b).

Distance to forest edge, elevation, slope, population density, and distance to water channels were identified as the major contributors to local mammalian species richness (Table 3). These five variables collectively accounted for 53% of the total relative importance. In particular, in 10 out of 16 surveyed species, distance to the forest edge and elevation were crucial factors (i.e., within the top 3) influencing their distribution. In contrast, bioclimate variables were not important factors affecting mammalian species.

**Figure 3:**
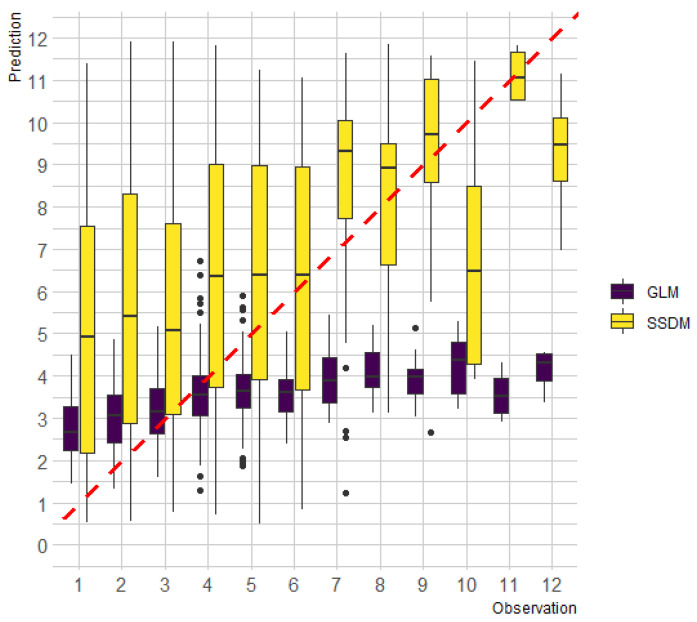
Comparison of mammalian species richness prediction between the stacked species distribution model (SSDM) and a macroecological model (GLM, i.e., Poisson regression). The red dashed line represents a 1:1 line.

The difference between predicted and observed species richness, denoted as species richness error, averaged 3.89 (Table 4), indicating that the SSDM somewhat lacked precision for the prediction of mammalian species richness. However, the metrics of the proportion of correct predictions (0.72) and sensitivity (0.93) indicated that the performance of the model in detecting the presence of mammalian species was notable. Compared to a macroecological model, the overprediction of species richness by SSDM tended to occur at the sampling points with lower species richness. In contrast, superior performance was demonstrated at sampling points with higher species richness (Figure 3).

## 4. Discussion

The modeling results indicating that the distance to the forest edge is the most important variable influencing mammalian richness in Chungnam were not unexpected. This result supports other findings; for example, Rovero et al. [33] reported that the distance to the forest edge is the major determinant for the distribution of most tropical mammalian species. In the context of fragmented forests, such as our study area, the impact of the distance to the forest edge on species richness may be more pronounced [34].

This speculation is reinforced by our observations of a decline in species richness with increasing distance to the forest edge when using both SSDM and the macroecological model (Figure S1a and Table S1). The prevalence of generalist species that have been observed among species that are highly affected by the distance to the forest edge might indicate that the long history of forest fragmentation in the study area [21] might be a factor in the dominance of matrix-tolerant mammals. According to Laurance [35], those species may have advantages in (1) dispersing between fragments (rescue effect), (2) recolonizing fragments against local extinctions, and (3) exploitation of resources such as food and habitat around fragments (edge effect).

The relationship between biodiversity and elevation has been studied extensively in the fields of ecology and biogeography [36,37]. Considering the hump-shaped pattern of species richness with elevation [38,39], the peak of species richness would be expected to occur at intermediate elevation levels (Mid-domain effect; *sensu* [40]). This effect explains the increasing pattern observed in the mid-elevational zone, which can be attributed in part to the increased overlapping of species’ random placements among hard boundaries such as rivers and summits at the mid-point of the domain, even without any ecological or evolutionary processes [40,41]. If so, the continuously increasing species richness with increasing elevation (Figure S1b) might indicate that the topography of the study area is adequately gentle, allowing for a broad range of overlapping habitat types.

While preferences for slope gradients may differ for each wildlife species, a common preference for gentle slopes can be observed in many mammalian species in Korea [42], including Siberian roe deer (*Capreolus pygargus*) [43], leopard cat [17], Korean water deer [15,44], and wild boar (*Sus scrofa*) [45]. Compared to other neighboring provinces, the adequate forests with gentle slope gradients found within the study region can serve as ecological corridors, facilitating movement [44], enhancing the availability of water [43] and food resources [44], providing sites for resting and hunting [46], and for avoiding mountainous carnivores [15] and human conflicts [17].

The growing human population and subsequent activities have led to the shift from a mosaic of diverse ecosystems to a uniform landscape with human-dominated ecosystems [47]. The impact of humans on species diversity has been well-documented, as human population density has been reported as a major cause of the decline of species diversity, including mammalian species [48]. The observed tendency toward a negative association between population density and mammalian richness (Figure S1d) corresponds with this assertion. However, the relationship is still being debated, and the existing documented studies focus primarily on sensitive individual species; thus, caution is warranted in drawing a general conclusion [49].

Proximity to water, in conjunction with food sources and green vegetation cover, has been reported as a critical factor in shaping species distribution [50,51]. However, the low relative importance value for the distance to water may be attributed in part to the scale of the study region. Rich et al. [52] suggested that water might be less of a limiting factor on a regional scale. In addition, given the generally moist and mild climate and abundant water resources within the study region, water is not considered a limiting factor for wildlife habitat [53]. Additionally, for certain prey species, maintaining distance from water may be an adoptable strategic behavior to avoid predation, as suggested by de Boer et al. [54].

However, caution is warranted when interpreting SSDM results due to several limitations. First, as with all other species distribution modeling approaches, incomplete species occurrence data and imperfect model specification can result in increased uncertainty and bias [55]. Discrepancies in resolutions and mismatches among environmental variables used in fitting the ESDMs (for detail, please refer to Table S2) may not be a sound representation of the actual conditions experienced by each organism [56]. Furthermore, the environmental variables identified by SSDM do not account for the positive or negative relationships to individual species. As a result, the magnitudes of effects of environmental variables on species richness could be different from those predicted by SSDM.

Overestimation has also been a criticism of SSDM ([12,30,57]), and the results of our study support these concerns, showing overestimation in areas where low species richness has been observed. Earlier studies (e.g., [11,58,59]) reported on the impact of species prevalence using biased results. In addition, the scale of the study region may be another factor affecting model performance, as relationships between species richness and environmental effects can vary according to spatial scale [13]. Lack of consideration for biotic interaction might also be a potential contributor to overestimation [60].

Nonetheless, the results of the study demonstrated the usefulness of the SSDM in the management of wildlife in Chungnam. Its superior performance, when compared with the macroecological approach in areas where high species richness was observed, was demonstrated. It was able to identify environmental variables that are important for mammalian species richness, which were not identified when using the macroecological model. The discrepancy in results between the SSDM and the macroecological approach might indicate the necessity of using an ensemble modeling approach. In addition, the strong correlation between species richness and endemism shown by the SSDM (Figure 2) might indicate that areas with an abundance of endemic species should be given higher priority for enhancing and conserving overall species richness. Therefore, the findings of this study can provide valuable insights that may be helpful to land managers in the effort to facilitate better decision-making for effective management of wildlife habitats.

## 5. Conclusions

Based on the results of SSDM modeling, significant environmental variables influencing regional mammalian species richness in Chungnam were identified. The distance to the forest edge, which was the most influential variable, would presumably be more pronounced in fragmented forests with a long history of land use. The negative association between the distance to the forest edge and mammalian species richness might indicate that forest fragmentation supports matrix-tolerant mammals. The model indicated that the gentle topography (i.e., elevation and slope) is also a critical environmental variable affecting mammalian species richness in the study region. Human impacts appear to have a negative impact on mammalian species richness, whereas bioclimate variables were not a limiting factor for species richness. Overestimation, when using the SSDM, cautions that species prevalence, spatial scale, and biotic interaction should be considered when interpreting modeling results. Despite these challenges, the findings of the study demonstrated that valuable information and insights can be obtained with use of the SSDM, which can be utilized by land managers for effective management of wildlife habitats, particularly in areas with high species richness and an abundance of endemic species.

## Figures and Tables

**Table 1:** Description of variables for model construction.

Variables	Notation	Unit	Note	Source ^†^
Mammal Occurrence	-	-	Binomial	CI
Forest Area	F_AREA	m^2^		KFS
Diameter Class	DIA_CL	-	Categorical data	KFS
Distance to Forest Edge	DIST_FOR	m	Log-transformed	KFS
Distance to Water Channel	DIST_WAT	m	Log-transformed	KNGII
Elevation	ELEV	m		KNGII
Slope	SLOPE	%		KNGII
Population Density	POP_DEN	people km^−2^	Log-transformed	KNGII
Road Density	RD_DEN	roads km^−2^		KNGII
Annual Mean Temperature	BIO1	°C		WorldClim
Mean Diurnal Range	BIO2	°C		WorldClim
Isothermality	BIO3	%	BIO2/BIO7 × 100	WorldClim
Temperature Seasonality	BIO4	°C	Standard deviation × 100	WorldClim
Max Temperature of Warmest Month	BIO5	°C		WorldClim
Min Temperature of Coldest Month	BIO6	°C		WorldClim
Temperature Annual Range	BIO7	°C	BIO5-BIO6	WorldClim
Mean Temperature of Wettest Quarter	BIO8	°C		WorldClim
Mean Temperature of Driest Quarter	BIO9	°C		WorldClim
Mean Temperature of Warmest Quarter	BIO10	°C		WorldClim
Mean Temperature of Coldest Quarter	BIO11	°C		WorldClim
Annual Precipitation	BIO12	mm		WorldClim
Precipitation of Wettest Month	BIO13	mm		WorldClim
Precipitation of Driest Month	BIO14	mm		WorldClim
Precipitation Seasonality	BIO15	-	Coefficient of variation	WorldClim
Precipitation of Wettest Quarter	BIO16	mm		WorldClim
Precipitation of Driest Quarter	BIO17	mm		WorldClim
Precipitation of Warmest Quarter	BIO18	mm		WorldClim
Precipitation of Coldest Quarter	BIO19	mm		WorldClim

^†^ CI: Chungnam Institute; KFS: Korea Forest Service; KNGII: Korea National Geographic Information Institute; WorldClim: WorldClim Database Bioclimate Variables (ver. 2.1).

**Table 2:** Mammalian species identified during the field campaign (*n* = 1357). The nomenclature of species names followed the Database of the National Species List of Korea.

Species Name	Scientific Name	Notation	Occurrences
Korean water deer	*Hydropotes inermis*	HYIN	1209
Large mole	*Mogera robusta*	MORO	858
Eurasian red squirrel	*Sciurus vulgaris*	SCVU	675
Common raccoon dog	*Nyctereutes procyonoides*	NYPR	545
Leopard cat	*Prionailurus bengalensis*	PRBE	370
Siberian chipmunks	*Eutamias sibiricus*	EUSI	177
Yellow weasel	*Mustela sibirica*	MUSI	167
Korean hare	*Lepus coreanus*	LECO	136
Wild boar	*Sus scrofa*	SUSC	90
Eurasian river otter	*Lutra lutra*	LULU	76
Asian Badger	*Meles leucurus*	MELE	34
Amur hedgehog	*Erinaceus amurensis*	ERAM	24
Siberian roe deer	*Capreolus pygargus*	CAPY	23
Yellow-throated marten	*Martes flavigula*	MAFL	10
Eurasian harvest mouse	*Micromys minutus*	MIMI	10
Siberian flying squirrel	*Pteromys volans*	PTVO	5

**Table 3:** Relative importance of the top 10 variables affecting mammalian species richness and species whose distribution is impacted by those variables. Species were listed if each species’ distribution model indicated that the relative importance (parenthesis) of the variable was within the top three variables. Notations for variable and species are described in Table 2 and Table 3, respectively.

Variable	Relative Importance	Impacted Species
DIST_FOR	28.4	EUSI (16.8), HYIN (74.6), LECO (19.4), LULU (17.5), MORO (78.6), MUSI (43.7), NYPR (51.6), PRBE (54.2), SCVU (62.5), SUSC (7.9)
ELEV	10.1	CAPY (13.8), ERAM (12.2), EUSI (12.8), HYIN (5.5), MAFL (9.5), MELE (13.3), MUSI (9.3), NYPR (5.4), PRBE (5.6), SUSC (50.5)
SLOPE	5.8	CAPY (9.7), ERAM (16.0), LECO (12.4), LULU (10.9), MAFL (7.8), MORO (2.9)
POP_DEN	4.9	MAFL (15.3), MELE (11.6)
DIST_WAT	4.1	LECO (11.7), MELE (13.4)
DIA_CL	3.7	CAPY (16.9), LULU (7.5), MORO (1.6)
BIO10	3.3	ERAM (22.1)
BIO13	2.9	-
F_AREA	2.7	SUSC (4.6)
BIO4	2.7	EUSI (6.6), SCVU (4.3)

**Table 4:** Model evaluation matrix for the stacked species distribution model for mammals in the Province of Chungnam.

Species Richness Error	Prediction Success	Cohen’s Kappa	Specificity	Sensitivity	Jaccard Index
3.89	0.72	1.00	0.67	0.93	0.41

## Data Availability

The data presented in this study were obtained from various entities, as shown in Table 1. The data may be available from the entities upon reasonable request.

## Supplementary Materials

The following supporting information can be downloaded at: https://www.mdpi.com/article/10.3390/ani14050759/s1. Figure S1: Scatter plots between observed species richness and (a) the distance to forest edge, (b) elevation, (c) slope, and (d) population density, respectively. Red lines present the loess smoothing lines.; Table S1: Summary statistics of the macroecology model (Poisson regression) for mammalian species richness in the Province of Chungman. Notations for the variables were described in Table 2.; Table S2: Summary statistics of ensemble species distribution model performance for individual mammalian species in the Province of Chungman. Notations for the species were described in Table 3.
