# Supplementary material for: Assessing the Distribution and Richness of Mammalian Species Using a Stacking Species Distribution Model in a Temperate Forest
Source: Animals (Basel). 2024 Feb 29;14(5):759. doi: 10.3390/ani14050759 (PMC10930543; doi:10.3390/ani14050759)
Supplement: Supplementary file 1 [file animals-14-00759-s001.zip › animals-2845275-supplementary.pdf]

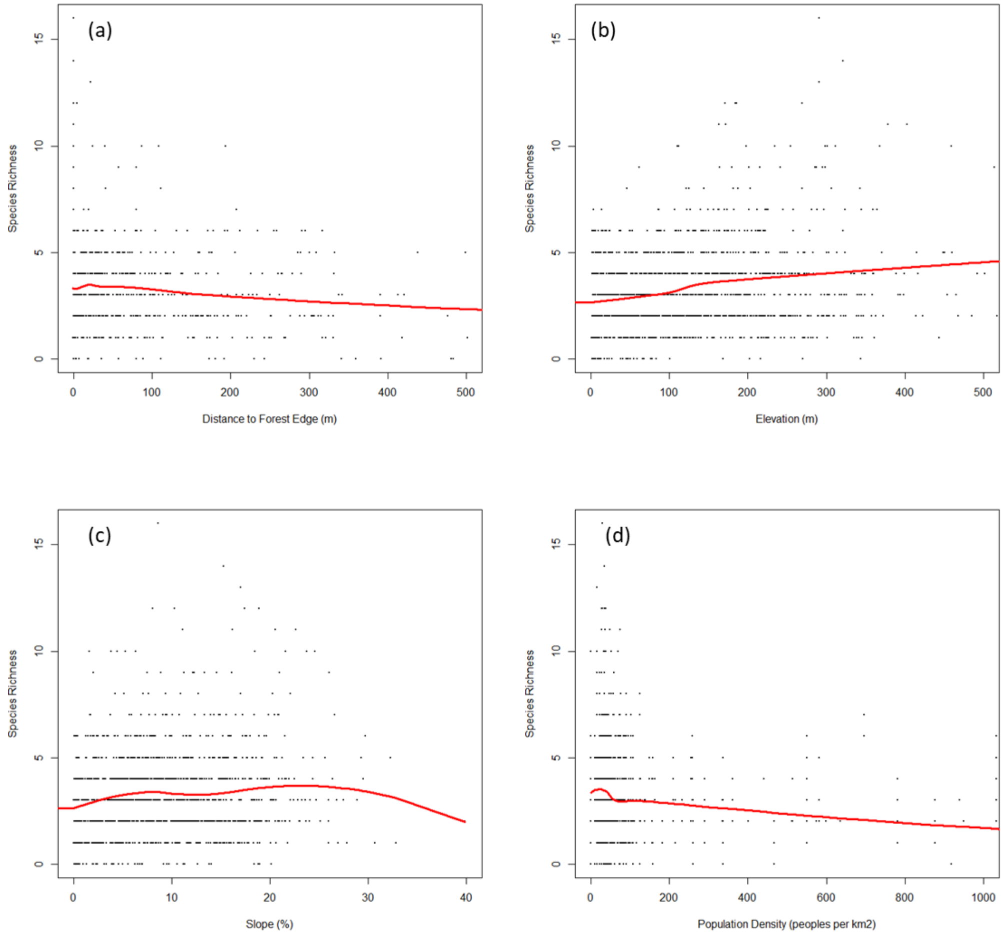

Figure S1. Scatter plots between observed species richness and (a) the distance to the forest edge, (b) elevation, (c) slope, and (d) population density, respectively. Red lines present the loess smoothing lines.

Table S1. Summary statistics of the macroecology model (Poisson regression) for mammalian species richness in the Province of Chungman. Notations for the variables are described in Table 2.

Variable	Estimate	Std. Error	z value	Pr(>|z|)
(Intercept)	2.18E+01	4.35E+00	5.002	5.68E-07
DIST_WAT	-2.52E-05	9.27E-06	-2.715	0.00664
DIST_FOR	-2.44E-04	1.25E-04	-1.95	0.05115
BIO2	2.19E+00	3.63E-01	6.047	1.48E-09
BIO3	-6.65E-01	9.12E-02	-7.293	3.03E-13
BIO4	-1.39E-01	2.70E-02	-5.169	2.35E-07
BIO7	-7.86E-01	2.88E-01	-2.727	0.0064
BIO9	-6.08E+00	9.21E-01	-6.597	4.19E-11
BIO10	5.45E+00	9.43E-01	5.775	7.70E-09
BIO12	2.79E-02	4.09E-03	6.824	8.87E-12
BIO13	-9.66E-02	1.18E-02	-8.219	< 2e-16
BIO14	-6.92E-02	2.21E-02	-3.131	0.00174
BIO15	1.83E-01	4.46E-02	4.116	3.85E-05
BIO16	-1.65E-02	5.36E-03	-3.077	0.00209

Table S2. Summary statistics of ensemble species distribution model performance for individual mammalian species in the Province of Chungman. Notations for the species are described in Table 3.

Species	Algorithm	Threshold	AUC	Sensitivity	Specificity	Cohen's kappa
CAPY	GLM	0.039	0.80	0.80	0.80	0.09
	RF	0.525	1.00	1.00	1.00	1.00
	CTA	0.500	0.70	0.40	1.00	0.40
	SVM	0.534	1.00	1.00	1.00	1.00
ERAM	GLM	0.072	0.97	1.00	0.95	0.38
	GBM	0.203	0.80	0.80	0.80	0.60
	RF	0.366	0.80	0.80	0.80	0.60
	CTA	0.500	0.90	1.00	0.80	0.80
	SVM	0.641	0.80	0.80	0.80	0.60
EUSI	GLM	0.186	0.76	0.76	0.76	0.31
	GBM	0.270	0.84	0.84	0.84	0.68
	RF	0.672	0.73	0.74	0.73	0.47
	CTA	0.045	0.73	0.74	0.73	0.47
	SVM	0.652	0.76	0.76	0.76	0.52
HYIN	GLM	0.537	0.67	0.67	0.67	0.33
	GBM	0.461	0.66	0.65	0.66	0.31
	RF	0.544	0.70	0.70	0.70	0.40
	CTA	0.474	0.72	0.86	0.58	0.45
	SVM	0.821	0.70	0.71	0.70	0.41
LECO	GBM	0.001	0.74	0.70	0.78	0.48
	RF	0.539	0.83	0.81	0.85	0.66
	CTA	0.751	0.70	0.81	0.59	0.41
	SVM	0.708	0.74	0.74	0.73	0.47
LULU	CTA	0.250	0.75	0.67	0.83	0.50
	SVM	0.533	0.76	0.75	0.77	0.52
MALF	GBM	0.170	0.75	0.50	1.00	0.50
	RF	0.470	1.00	1.00	1.00	1.00
	CTA	0.500	0.75	0.50	0.00	-0.50
MELE	GLM	0.045	0.72	0.71	0.72	0.07
	GBM	0.078	0.79	0.71	0.86	0.57
	RF	0.596	0.86	0.86	0.86	0.71
	SVM	0.661	0.71	0.71	0.71	0.43
MIMI	GLM	0.019	0.91	1.00	0.83	0.06
	RF	0.443	1.00	1.00	1.00	1.00
	CTA	0.500	1.00	1.00	1.00	1.00
MORO	GLM	0.440	0.71	0.71	0.71	0.39
	GBM	0.360	0.72	0.72	0.72	0.45
	RF	0.576	0.76	0.76	0.75	0.51
	SVM	0.762	0.74	0.74	0.74	0.49
MUSI	GLM	0.150	0.77	0.77	0.78	0.27
	GBM	0.201	0.65	0.65	0.65	0.31
	RF	0.475	0.85	0.85	0.85	0.69
	CTA	0.500	0.65	0.69	0.62	0.31

	SVM	0.627	0.85	0.85	0.85	0.69
NYPR	GLM	0.294	0.71	0.71	0.70	0.34
	GBM	0.445	0.75	0.75	0.75	0.50
	RF	0.583	0.69	0.69	0.69	0.38
	CTA	0.626	0.69	0.69	0.68	0.37
	SVM	0.831	0.73	0.73	0.73	0.45
PRBE	GLM	0.278	0.69	0.70	0.69	0.28
	GBM	0.447	0.74	0.74	0.74	0.47
	RF	0.535	0.79	0.80	0.79	0.59
	CTA	0.129	0.70	0.68	0.72	0.41
	SVM	0.575	0.72	0.72	0.72	0.45
PTVO	GLM	0.027	0.98	1.00	0.96	0.14
	RF	0.597	1.00	1.00	1.00	1.00
	CTA	0.500	1.00	1.00	1.00	1.00
	SVM	0.905	1.00	1.00	1.00	1.00
SCVU	GLM	0.390	0.74	0.74	0.73	0.42
	GBM	0.309	0.78	0.78	0.78	0.57
	RF	0.522	0.83	0.83	0.83	0.66
	CTA	0.576	0.72	0.72	0.72	0.43
	SVM	0.684	0.79	0.79	0.79	0.58
SUSC	GLM	0.108	0.75	0.75	0.74	0.19
	GBM	0.440	0.79	0.80	0.79	0.59
	RF	0.454	0.80	0.80	0.80	0.60
	SVM	0.677	0.74	0.75	0.74	0.49
